# Foodborne Pathogen Assessment in Raw Milk Cheeses

**DOI:** 10.1155/2020/3616713

**Published:** 2020-01-22

**Authors:** Nicola Costanzo, Carlotta Ceniti, Adriano Santoro, Maria Teresa Clausi, Francesco Casalinuovo

**Affiliations:** ^1^Department of Health Sciences, University “Magna Græcia” of Catanzaro, Campus Universitario “S. Venuta”, Viale “S. Venuta”, I-88100 Catanzaro, Italy; ^2^Department of Veterinary Medicine and Animal Production, University “Federico II” of Naples, Via Delpino 1, 80100 Napoli, Italy; ^3^Istituto Zooprofilattico Sperimentale del Mezzogiorno, Viale Crotone, Catanzaro 88100, Italy

## Abstract

General hygienic parameters and selected foodborne pathogens in raw milk cheeses at the retail level were evaluated. A total of 245 raw milk cheese samples were analysed for total bacterial count, *Enterobacteriaceae*, *E. coli*, *Salmonella* spp., *Listeria monocytogenes*, *coagulase-positive Staphylococci*, and staphylococcal enterotoxin. Results showed only 3 samples that were not compliant with European rules on staphylococcal enterotoxin, but *coagulase-positive Staphylococci* were evidenced in all samples tested. *Salmonella* spp. and *Listeria monocytogenes* were never detected whereas *E. coli* was evidenced in 20 samples. Results suggest a need for improvement of good manufacturing practice and milking operation.

## 1. Introduction

In recent years, small-scale artisanal cheese production has increased worldwide and in Italy too. Raw milk is becoming frequently used in cheese production and gaining popularity among consumers who recognize raw milk cheese as healthier and as a better representative of the local food tradition. Unpasteurized milk allows the natural enzymes and microflora proteolysis and lipolysis activity to enhance desirable flavour characteristics [1]. Growth of pathogens is hindered by several factors such as speed of curd acidification and final product pH; time and temperature used to rinse the curd; the presence in milk of natural protection systems such as lactoperoxidase, lysozyme, and lactoferrin, well known for their antibacterial activity [2]; and production of bacteriocins or a bacteriocin-like compound by lactic acid bacteria [3]. Additionally, the cheese made from milk that has not undergone heat treatment may represent a food safety concern especially for consumers under special medical and physiological-modified conditions. Although pathogens like *Salmonella*, *Listeria monocytogenes*, verotoxigenic *Escherichia* O157, and *Staphylococcus aureus* are often part of the animal intestinal microbiota, they can be isolated from the udder of healthy dairy animals or from operators' hands, and thus, they can easily contaminate the milk during the milking process [4].

*Listeria monocytogenes* represents one of the main concerns for ready-to-eat (RTE) food including raw milk cheeses, due its high mortality rate [5] especially for individuals at higher risk, e.g., pregnant women and immunocompromised people. In 2017, EFSA reported 2480 [6] confirmed invasive human cases of listeriosis and a prevalence rate of 0.9% in soft and semisoft cheeses made from raw or low-heat-treated milk. *Listeria monocytogenes* presence in raw milk and cheese has been widely reported [7], and its ubiquitous nature and distribution contamination may occur at any stage of the production chain.

*Salmonella* spp. still represents the first causative agent of foodborne outbreak in EU: 9600 human cases, 2227 hospitalisations. *Salmonella*, the causative agent of foodborne outbreaks associated with the consumption of various types of cheese [8], can be detected in the intestinal tract of healthy animals, and contamination of milk mainly occurs during milking operations [9].

*Staphylococcus aureus*, cause a typical food poisoning which is characterized by nausea, vomiting, and diarrhoea [10, 11], and it is linked to consumption of food contaminated by one or more preformed enterotoxins [11] that are produced when the bacterial load exceeds 10^5^ CFU/g [12]. Staphylococcal food poisoning due to soft cheese made from raw milk has been recently reported [13].

*E. coli* occurrence in raw milk, in particular serotype 0157-H7, is well documented and represents a concern since up to 10% of people infected with these bacteria develop haemolytic uremic syndrome (HUS), which is potentially a lethal condition, especially in children [14]. In 2018, EFSA reported a total of 5079 foodborne outbreaks of which 14, involving 775 people, were linked to cheese consumption, and the most common causative agents were Shiga toxin-producing *E. coli* (STEC) and bacterial toxin [6].

Food safety criteria for staphylococcal enterotoxins, *Salmonella*, and *Listeria monocytogenes* in cheeses made from raw milk or milk that has undergone a lower heat treatment than pasteurization are laid down in Regulation 1441/2007 [15].

The present study was aimed at assessing the prevalence of target pathogens and enterotoxins in raw milk cheese samples collected at the retail level in Italy.

## 2. Materials and Methods

### 2.1. Sample Collection

From January to December 2018, a total of 245 raw milk cheese samples, singularly packaged from the manufacturer, were collected from local markets located in Italy (Calabria region). Among those, 182 originated from ovine, 27 from bovine, and 36 were produced using a mixture of bovine and ovine milk. Ovine cheese samples were categorized into the two following groups: the hard cheese category (80 samples) with a ripening period ≥ 60 days and the soft cheese category (102 samples) with a ripening period of ≤60 days. Bovine cheese samples consisted in two different categories: the soft cheese category (18 samples) and the pasta filata cheese category (9 samples). All mixed milk cheese samples (36 samples) belonged to the soft cheese category. Following collection, cheese samples were kept at 4°C and they were transported to the laboratory where analysis was carried out within 4 hours.

### 2.2. Sample Preparation

For each sample, an amount of 25 g of cheese was removed with a sterile blade, placed into a sterile stomacher bag, and subsequently homogenized in 225 mL of a quarter-strength Ringer solution (BR0052, Oxoid Ltd., Hampshire, UK) for 2 minutes using a Stomacher (Seward Laboratory Stomacher 400 Lab Blender, UK). Tenfold dilutions per homogenate sample were performed.

### 2.3. Total Viable Counts and *Enterobacteriaceae* and *Escherichia coli* Enumeration

Total bacterial count (TBC) and *Enterobacteriaceae* and *E. coli* enumeration were performed by the culture method. Thus, one mL from dilution was inoculated onto Plate Count Agar (PCA, CM0325, Oxoid Ltd.) and incubated for five days at 32°C, onto Violet Red Bile Glucose Agar (VRBGA, CM1082, Oxoid Ltd.) and incubated for 24 h at 37°C, and onto RAPID *Escherichia* agar (Bio-Rad, Reinach, Switzerland) for 24 h at 37°C for TBC, *Enterobacteriaceae* enumeration, and *E. coli* enumeration, respectively.

### 2.4. *Staphylococcus* Coagulase-Positive Enumeration and Enterotoxin Detection

For *Staphylococcus* coagulase-positive (SCP) enumeration, 0.1 mL from each dilution was spread onto Baird-Parker+Rabbit Plasma Fibrinogen according to ISO 6888-1/2 (2004) and incubated at 37°C for 48 h. Following the incubation period, five colonies per plate were tested for Gram coloration, catalase (Sigma-Aldrich, St. Louis, USA), coagulase (Thermo Fisher, Waltham, USA), and thermonuclease (Sigma-Aldrich, St. Louis, USA) and then phenotypically identified by the API Staph system (bioMérieux, Marcy-l'Étoile, France).

The presence of enterotoxin was assessed using the automated VIDAS Staph enterotoxin II, SET 2 (bioMérieux, Marcy-l'Étoile, France) based on an enzyme-linked fluorescent assay according to the instructions of the manufacturer.

### 2.5. *Salmonella* Detection

*Salmonella* analyses were done in accordance with ISO 6579:09 using a two-step enrichment procedure. Briefly, 10 mL of cheese from each sample was preenriched in 90 mL of Buffered Peptone Water (BPW, CM1049, Oxoid Ltd.) for 24 h at 37°C. Then, 1 mL of the preenriched broth was transferred into 10 mL of Muller-Kauffmann Tetrathionate-Novobiocin Broth (CM1048, Oxoid Ltd.) supplemented with Novobiocin Selective Supplement (SR0181, Oxoid Ltd.) according to the manufacturer's instructions and incubated for 24 h at 37°C; moreover, 0.1 mL of the preenriched broth was also inoculated onto 10 mL of Rappaport-Vassiliadis Soya Peptone Broth (CM0866, Oxoid Ltd.) and incubated for 24 h at 41.5°C. Subsequently, each enriched broth was spread onto different selective media of Mannitol Lysine Crystal Violet Brilliant Green Agar (MLCB Agar, CM0783, Oxoid Ltd.) and onto Xylose-Lysine-Desoxycholate Agar (XLD Agar, CM0469, Oxoid Ltd.) using a sterile loop and incubated for 24 h at 37°C. Colonies were identified using the API 20E system (bioMérieux, Marcy-l'Étoile, France) according to the manufacturer's indications.

### 2.6. *Listeria monocytogenes* Detection

In accordance with ISO 11290-1:2004, detection of *L. monocytogenes* was carried out by suspending 10 g of cheese in 90 mL of Fraser Broth (CM0895, Oxoid Ltd.) with Half Fraser Supplement (SR0166, Oxoid Ltd.) and incubating for 24 h at 30°C. Subsequently, 0.1 mL of the first enrichment broth was suspended in 10 mL of Fraser Broth (CM0895, Oxoid Ltd.) with Fraser Supplement (SR0156, Oxoid Ltd.) and then incubated for 24 h at 37°C. One loopfull of the enrichment broth was then streaked onto PALCAM Agar (CM0877, Oxoid Ltd.) added with PALCAM Selective Supplement (SR0150, Oxoid Ltd.) and onto Chromogenic Listeria Agar (CM1084, Oxoid Ltd.) supplemented with Listeria Selective Supplement (SR0226, Oxoid Ltd.) and Listeria Differential Supplement (SR0244, Oxoid Ltd.). Both plates were incubated for 48 h at 37°C, then *Listeria monocytogenes* presumptive colonies were biochemically identified by using the API Listeria System according to manufacturer's instructions (bioMérieux).

## 3. Results

The results of TBC, reported in Table 1, showed a high bacterial load in all samples tested. Soft cheese samples made from ovine milk evidenced the highest TBC level since more than 50% (*n* = 60) of the samples analysed showed a TBC greater than 6 log CFU/g; in the same category, hard cheese showed a lower contamination rate since more than 50% of the samples analysed (*n* = 43) showed a TBC count below 6 log CFU/g. In cheese made from bovine milk, pasta filata cheeses samples showed the lowest TBC range: enumeration in all samples ranged between 5 and 6 log CFU/g differently from hard cheese samples where a TBC level above 6 log CFU/g was evidenced in all samples tested. Overall, cheese from bovine milk showed lower TBC enumeration compared to ovine and mixed milk cheeses.

The results of *Enterobacteriaceae* enumeration are reported in Table 2. Out of 245 cheese samples, 25 resulted positive for *Enterobacteriaceae*, and among these, *E. coli* was evidenced in 20 samples.

With regard to cheese category and milk origin, ovine milk soft cheeses resulted in the highest number of *E. coli*-positive samples. Out of the 20 *E. coli*-positive samples, 10 belonged to the soft cheese category, all made from ovine milk, and 10 to hard cheese: 4 were produced from ovine milk, 1 was produced from bovine milk, 3 were produced from a mix of ovine and bovine milk cheese, and 2 were from a mix of goat and ovine cheese. Additionally, in all samples positive for *Enterobacteriaceae* and *E. coli*, the contamination rate was very similar suggesting that most of the *Enterobacteriaceae* were *E. coli*.

SCP were isolated in all samples analysed: the average count was 2.52 log UFC/g. Out of 245 samples analysed, 55 showed a SCP contamination level of 5∗10^5^: 37 were cheese made from ovine milk (25 soft cheese and 12 hard cheese), 13 were cheese made from bovine milk (pasta filata), and 5 were from mixed milk cheese. All samples exceeding the 5 log threshold level, specified in Regulation (EC) 1441/2007, were tested for enterotoxin: 3 samples, all cheeses made from ovine milk, were positive for enterotoxin and the isolates were identified as S. *aureus*, *S*. *epidermidis*, and *S*. *warneri*.

*Listeria monocytogenes* and *Salmonella* were never detected in all samples tested.

## 4. Discussion

Raw milk cheeses are an important part of the Italian cheese making tradition since productions are carried out in small and artisanal facilities strongly linked to the territory. The lack of the pasteurization step in cheese production preserves the indigenous bacteria responsible, through their enzymatic modification, for some important physical-chemical characteristic. Nevertheless, unpasteurized milk poses a microbiological risk since pathogens are not destroyed by the heat treatment. In this study, a microbiological evaluation of raw milk cheeses was carried out and it highlighted a high bacterial load in all the samples tested as reported in previous studies [16, 17]. Soft cheese, regardless of milk origin, showed in more than 50% of the samples analysed a total bacterial count above 6 log CFU/g whereas pasta filata cheese samples were all below 6 log CFU/g. This value might be due to the curd stretching step performed in hot water and it has been already reported for similar semihard pasta filata cheeses [18].

Results of *Enterobacteriaceae* and *E. coli* enumeration showed a good hygienic level in the samples tested: out of 245 samples analysed, 25 and 20 were positive for *Enterobacteriaceae* and *E. coli*, respectively. Soft cheese made from ovine milk was more often contaminated: out of 25 positive samples, 13 and 10 soft ovine cheese samples evidenced *Enterobacteriaceae* and *E. coli*, respectively. Despite the small number of positive samples, *Escherichia coli* presence has to be taken into account since it has been responsible of foodborne outbreaks caused by raw milk cheese consumption [19, 20]. *Escherichia coli* presence in raw milk cheese may indicate a milk contamination of faecal origin or can be linked to mammary infections. Additionally, ruminants are a reservoir for *E. coli* and defecation during milking is considered a critical event for milk contamination; therefore, good milking and hygiene practices must be maintained [21].

The high prevalence of SCP in raw milk cheese is well known [22], and our results are in agreement with other authors who reported a prevalence close to 100% in raw milk cheese [23] and at the retail level [24]. Nevertheless, our study showed that 42.86% of the retail samples analysed do not respond to the standards established by the EU, and moreover, 3 samples tested positive for enterotoxins. Despite *S. aureus* being one of the most common causes of mastitis in dairy animals [25] and one of the major foodborne pathogens worldwide [26], food poisoning outbreak linked to raw milk cheese consumption has been rarely reported [27]. Differently, it is generally believed that coagulase-negative *Staphylococci*, such as *S*. *epidermidis* and *S*. *warneri*, isolated in two positive samples, have low pathogenic potential. Our results are then in agreement with authors who have shown how coagulase-negative *Staphylococci* already represent a concern due to their ability to produce enterotoxin and to contain many virulence factors and antibiotic resistant genes [28]. *Staphylococci* can contaminate milk directly from clinical or subclinical mastitis but environment including human handling can also be a major contamination source; thus, improvements in production hygiene and selection of raw materials are strongly recommended.

In all tested samples, *Listeria monocytogenes* was never detected; this could be due to the presence of the endogenous microbiota of raw milk playing an inhibitive action on *L. monocytogenes* [29]. Beyond, listerial contamination may occur in many steps of the production chain: recently, no differences of prevalence between raw milk and pasteurized milk cheese have been reported [30] and postprocessing contamination still represents one of the major causes of cheese contamination with *L. monocytogenes* [29].

*Salmonella* has never been detected in all samples tested. Nevertheless, raw milk cheese has been recently linked to a *Salmonella* outbreak with 103 human cases linked to bovine raw milk cheese consumption [31].

## 5. Conclusion

Raw milk cheese still represents a concern in the EU including Italy where consumers are significantly more willing to buy raw milk cheeses than pasteurized milk cheese [32]. Raw milk cheeses, analysed in this study, can be considered as a low risk food due to the absence of major foodborne pathogens such as *Salmonella* and *Listeria monocytogenes*. The high prevalence of *Staphylococcus* and enterotoxin presence in 3 samples represent a concern and suggest a need for improvement of good manufacturing practice and milking operation; additionally, the improvement of the sanitary status of the herd is be desirable.

## Figures and Tables

**Table 1:** Total bacterial count of cheese samples according to cheese categories.

	Number of samples	Number and (%) of samples at different levels of TBC (log CFU/g)				
*Cheese made from ovine milk*		<5	>5 < 6	>6 < 7	>7 < 8	>8 < 9
Soft cheese	102	20 (19.6)	22 (21.6)	33 (32.4)	15 (14.7)	12 (11.7)
Hard cheese	80	11 (13.8)	32 (40)	12 (15)	18 (22.5)	7 (8.7)
*Cheese made from bovine milk*						
Hard cheese	18	1 (5.6)	6 (33.3)	8 (44.4)	2 (11.1)	1 (5.6)
Pasta filata	9	5 (55.6)	4 (44.4)	0 (0.0)	0 (0.0)	0 (0.0)
*Cheese made from mixed milk*						
Hard cheese	36	3 (8.2)	11 (30.6)	11 (30.6)	6 (16.7)	5 (13.9)

**Table 2:** *Enterobacteriaceae* and *E. coli* counts of cheese samples according to cheese categories.

	Number of samples	Number and (%) of samples at different levels of *Enterobacteriaceae*			Number and (%) of samples at different levels of *E.coli* (log CFU/g)	
*Cheese made from ovine milk*		<2	>2 < 3	>3 < 4	<2	>2 < 3
Soft cheese	102	8 (7.8)	3 (2.9)	2 (1.9)	6 (5.8)	4 (3.9)
Hard cheese	80	1 (1.3)	1 (1.3)	1 (1.3)	2 (2.5)	2 (2.5)
*Cheese made from bovine milk*						
Hard cheese	18	2 (11.1)	2 (11.1)	0 (0.0)	1 (5.5)	0 (0.0)
Pasta filata	9	0 (0.0)	0 (0.0)	0 (0.0)	0 (0.0)	0 (0.0)
*Cheese made from mixed milk*						
Hard cheese	36	2 (5.6)	3 (8.3)	0 (0.0)	2 (5.6)	3 (8.3)

## Data Availability

The quantitative data used to support the findings of this study are available from the corresponding author upon request, in excel database format.
